# Astrocyte-driven vasoconstriction impairs glymphatic clearance in a human tauopathy-on-chip model

**DOI:** 10.1063/5.0261875

**Published:** 2025-06-16

**Authors:** Rena Park, Yansong Peng, Aria R. Yslas, Esak Lee

**Affiliations:** Nancy E. and Peter C. Meinig School of Biomedical Engineering, Cornell University, Ithaca, New York 14853, USA

## Abstract

The glymphatic system is a critical pathway for clearing metabolic waste from the brain by mediating cerebrospinal fluid and interstitial fluid exchange. In Alzheimer's disease (AD), tau protein accumulation is strongly associated with impaired glymphatic clearance, yet the underlying mechanism remains poorly defined. In this study, we employed a three-dimensional human glymphatics-on-chip model to investigate fluid transport and mass clearance in a brain-mimetic extracellular matrix containing engineered blood vessels (BV) surrounded by primary astrocytes. We found that phosphorylated tau (p-tau) induced morphological transformation of astrocytes into a hypertrophic, hypercontractile state, leading to astrocyte-mediated vasoconstriction and impaired glymphatic clearance. Notably, p-tau did not affect blood endothelial cells directly, implicating astrocyte-dependent mechanisms in glymphatic deregulation. Pharmacological inhibition of non-muscle myosin II with blebbistatin reversed astrocytic hypercontractility, restored BV diameters, and rescued glymphatic function. These findings elucidate a glial-specific mechanism of tau-induced glymphatic dysfunction and underscore astrocytic contractility as a promising therapeutic target in AD.

## I. INTRODUCTION

The brain depends on a specialized waste clearance pathway, the glymphatic system, to maintain its homeostasis.^1,2^ This pathway directs the convective flow of the interstitial fluid (ISF) toward the cerebrospinal fluid (CSF) through the coordinated interaction of the perivascular space (PVS), astrocytic aquaporin-4 (AQP4) channels, and other cerebrovascular structures.^3,4^ Initially characterized in rodent models,^4,5^ the glymphatic pathway is now recognized in humans through imaging techniques such as intrathecal MRI,^6^ confirming the clinical relevance of this fluid transport system and highlighting its critical function in neurological health and disease.^1,7,8^ In Alzheimer's disease (AD), impaired glymphatic function is tightly linked to the accumulation of amyloid-β (Aβ) plaques and hyperphosphorylated tau (p-tau) proteins,^9–11^ both of which are the pathological hallmarks of AD.^12–14^ Tau is a microtubule-associated protein that aggregates into neurofibrillary tangles and paired helical filaments when hyperphosphorylated.^14,15^ Hyperphosphorylated tau enrichment in the brain parenchyma contributes to synaptic dysfunction, degeneration, and cognitive decline.^16–18^ While Aβ accumulation also disrupts glymphatic function,^19,20^ clinical studies have shown that the co-presence of Aβ and p-tau, rather than Aβ alone, poses stronger cognitive deterioration, suggesting that p-tau is another primary driver in AD progression.^21,22^

Although p-tau accumulation disrupts astrocytic AQP4 localization^23,24^ and glymphatic flow,^13^ the precise mechanisms by which tau affects the neurovascular unit (NVU) and impairs perivascular clearance remain unclear.^25,26^ Astrocytes, as key regulators of vascular tone and interstitial drainage,^27,28^ are believed to mediate early changes in cerebrovascular dynamics via the release of vasoactive molecules, such as prostaglandins, ATP, and nitric oxide.^17,24,29–31^ In particular, chronic cerebral hypoperfusion (CCH)—a common early feature of AD—has been associated with astrocyte reactivity and vascular remodeling.^32^ CCH is characterized by a sustained reduction in cerebral blood flow, often preceding cognitive symptoms and correlating with tau propagation and metabolic decline.^32,33^ While traditionally attributed to amyloid pathology or endothelial dysfunction,^34^ recent studies suggest that tau-associated astrocyte activation may contribute to diminished vascular support and blood–brain barrier integrity.^35^ In addition, cerebral hypoperfusion has been observed in the preclinical stages of AD, particularly among individuals with mild cognitive impairment, APOE4 genotype, or amyloid positivity.^33^ Despite these insights, the biomechanical role of astrocytes in regulating vascular tone in the context of tau pathology remains underexplored.^36^ This underscores the need to further elucidate glial contributions to early cerebrovascular dysfunction in AD.

Traditional experimental methods for inducing tau aggregation *in vitro* have frequently relied on artificial agents such as heparin to accelerate fibrillization.^37^ Tau species derived from these methods lack disease-specific post-translational modification, thereby having distinct aggregation profiles compared to human AD tissues.^38^ Recently, Meng *et al.* developed a sequential phosphorylation strategy involving protein kinase A (PKA) and stress-activated protein kinase 4 (SAPK3/MAPK12) and successfully generated tau species enriched for AD-relevant epitopes, including AT8, AT100, and PHF-1.^18^ PKA acts as a priming kinase that exposes downstream phospho-sites, while SAPK4 targets disease-associated residues. Importantly, tau phosphorylated by this method spontaneously aggregated into cytotoxic, amorphous structures and elicited TLR4-dependent inflammatory responses—recapitulating both the aggregation-prone and immune-activating features of pathological tau in AD. This kinase-based approach produces physiologically relevant tau aggregates without using non-physiological inducers, enabling the modeling of early astrocytic responses to pathogenic tau under conditions reflective of early-stage AD.

Several recent studies have advanced our understanding of tau-induced cerebrovascular dysfunction. Hussong *et al.* developed a two-dimensional *in vitro* cerebral endothelial cell model and found that exposure to oligomeric tau-induced oxidative stress, disrupted tight junction proteins, and increased blood–brain barrier (BBB) permeability through activation of the RhoA/ROCK signaling pathway.^25^ Similarly, Guzmán-Hernández and Fossati discovered that protofibrillar and fibrillar tau species promoted a glycolytic metabolic shift, endothelial inflammatory activation, and loss of barrier function in human cerebral microvascular endothelial cells.^39^ While these models have revealed that tau exposure can compromise endothelial barrier integrity and promote vascular inflammation,^25,39^ such approaches often overlook the role of astrocytes in vascular regulation,^40^ despite their critical function within the NVU. Moreover, two-dimensional models do not recapitulate interstitial fluid transport dynamics, and the contributions of tau pathology to glymphatic system impairment remain less well understood.

To address these gaps, we engineered a microfluidic-based glymphatics-on-chip model that incorporates primary human astrocytes and blood endothelial cells (BECs) within a three-dimensional (3D) brain-mimetic extracellular matrix (ECM).^41–43^ This platform enables multilayered investigation of neurovascular interactions and glymphatic fluid dynamics under physiologically relevant hydrostatic gradients while allowing for real-time functional readouts and longitudinal assessment of cellular responses. Our study reveals that astrocytic hypercontractility, triggered by p-tau, is sufficient to drive vasoconstriction and impair solute clearance. Importantly, this dysfunction is reversible with pharmacological inhibition of non-muscle myosin II, identifying astrocyte contractility as a potential therapeutic target in neurodegenerative disease.

## II. RESULTS

### A. Human glymphatics-on-chip model recapitulates 3D neurovascular architecture with astrocytic reactivity to tau

**FIG. 1.**
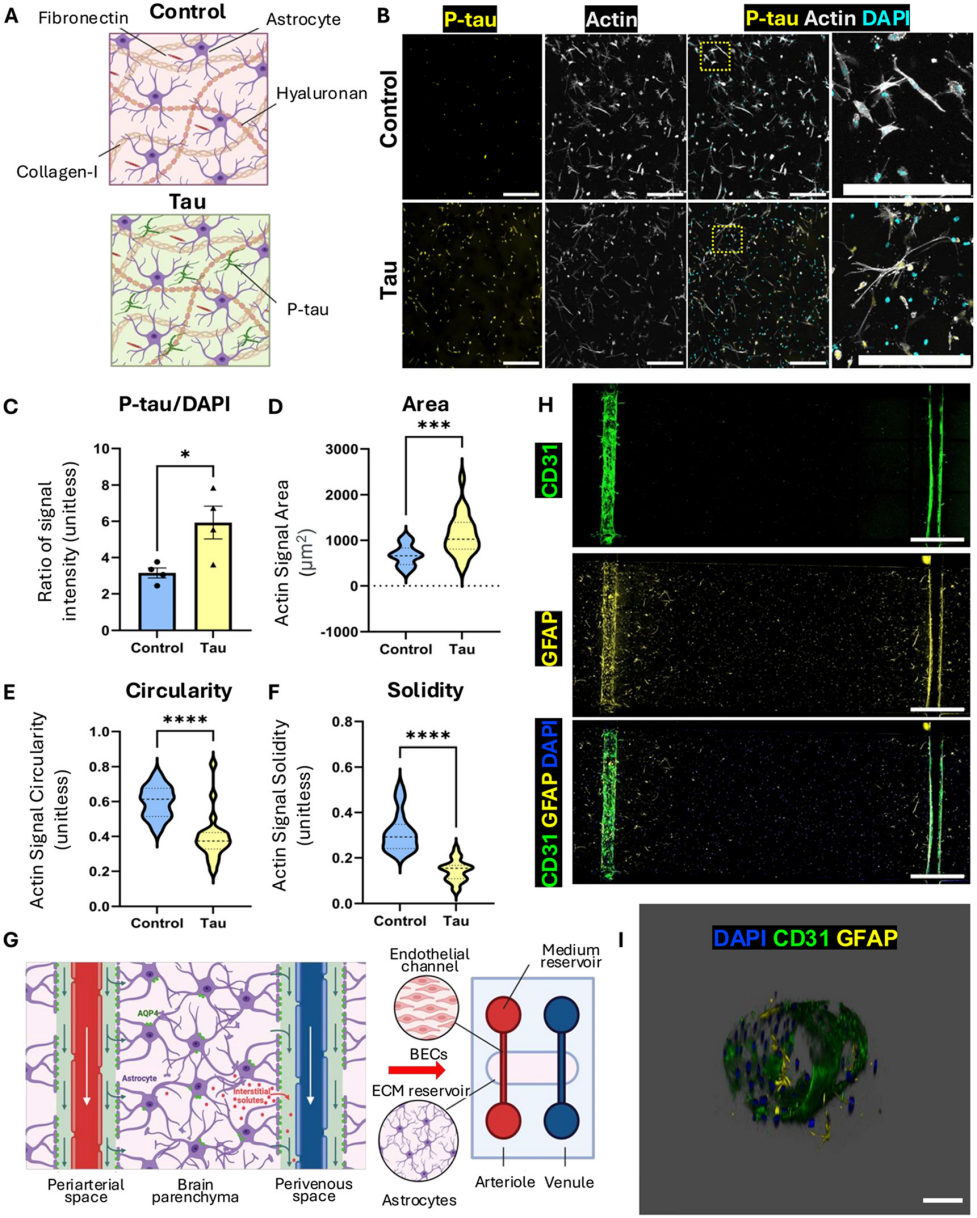
Human glymphatics-on-chip model recapitulates 3D neurovascular architecture with astrocytic reactivity to tau. (a) A schematic representation of the ECM components (astrocytes, fibronectin, collagen I, and hyaluronan) and experimental groups (control, red; tau-treated, green). (b) Immunofluorescence staining of phosphorylated tau (p-tau, yellow), actin (gray), and nuclei (DAPI, cyan) in control and tau-treated astrocytes. Zoomed insets highlight structural changes. (c) Quantification of p-tau signal intensity, normalized to DAPI (N = 4). (d) Violin plot showing astrocyte area under control and tau-treated conditions (N = 30–40). (e) Violin plot representing astrocyte circularity (N = 30–40). (f) Violin plot for astrocyte solidity, showing reduced compactness in tau-treated astrocytes (N = 30–40). (g) A schematic of the *in vivo* glymphatic system alongside the glymphatics-on-chip platform, depicting the arrangement of astrocytes and blood vessel channels. (h) Immunofluorescence images showing endothelial marker CD31 (green), astrocytic marker GFAP (yellow), and DAPI (blue) within the glymphatics-on-chip platform. (i) A representative, 3D-rendered blood vessel surrounded by astrocytes. Scale bars: 300 *μ*m (b), 1 mm (h), and 100 *μ*m (i). Statistical significance: ^*^(p < 0.05), ^***^(p < 0.001), and ^****^(p < 0.0001).

To mimic the native cerebral microenvironment,^43^ we developed a 3D matrix composed of collagen I, fibronectin, hyaluronan, and primary human cortical astrocytes [Fig. 1(a)]. Astrocytes were cultured with or without p-tau (1 *μ*M) at a physiologically relevant concentration derived from AD patient tissue data.^44^ To generate p-tau, we adopted a sequential kinase phosphorylation strategy using PKA and SAPK3/MAPK12 developed by Meng *et al.*^18^ This novel method yields AD-specific epitopes (AT8, AT100, and PHF-1) without relying on artificial aggregation agents like heparin,^37^ which allows us to capture early astrocytic responses to pathogenic tau under sub-cytotoxic, disease-relevant conditions.^38^ Immunofluorescence imaging revealed substantial morphological changes in tau-treated astrocytes [Fig. 1(b)]. Compared to controls, these cells exhibited increased p-tau accumulation [Fig. 1(c)], expanded actin-rich area [Fig. 1(d)], and a transformation from stellate to hypertrophic morphologies marked by decreased circularity and solidity [Figs. 1(e) and 1(f)]. These reactive features mimic astrocyte activation in early-stage AD.^45^ While not all cells appeared uniformly affected, a substantial subset of tau-treated astrocytes exhibited a tufted morphology with enlarged processes, highlighting the localized changes in their morphology and cytoskeletal structure.

Next, we integrated the brain-mimic ECM into a microfluidic platform engineered to support perfusable, BEC-lined channels [Figs. 1(g)–1(i)]. This dual-channel setup allowed us to visualize astrocyte-endothelial organization within a perfusable network, enabling functional simulation of the PVS [Fig. 1(g)]. Immunofluorescence staining with CD31 (endothelial marker) and GFAP (astrocytic marker) provided precise visualization of the perivascular and vascular architecture within the chip [Fig. 1(h)]. In particular, the platform enabled a perfusable and lumenized vasculature, which is critical to perform interstitial fluid drainage experiments [Fig. 1(i)]. These results confirm that the model successfully reconstructed perivascular architecture with distinguishable astrocytic and endothelial compartments, validating the platform's utility for glymphatic clearance studies.

### B. Tau-induced astrocytic reactivity drives blood vessel constriction

**FIG. 2.**
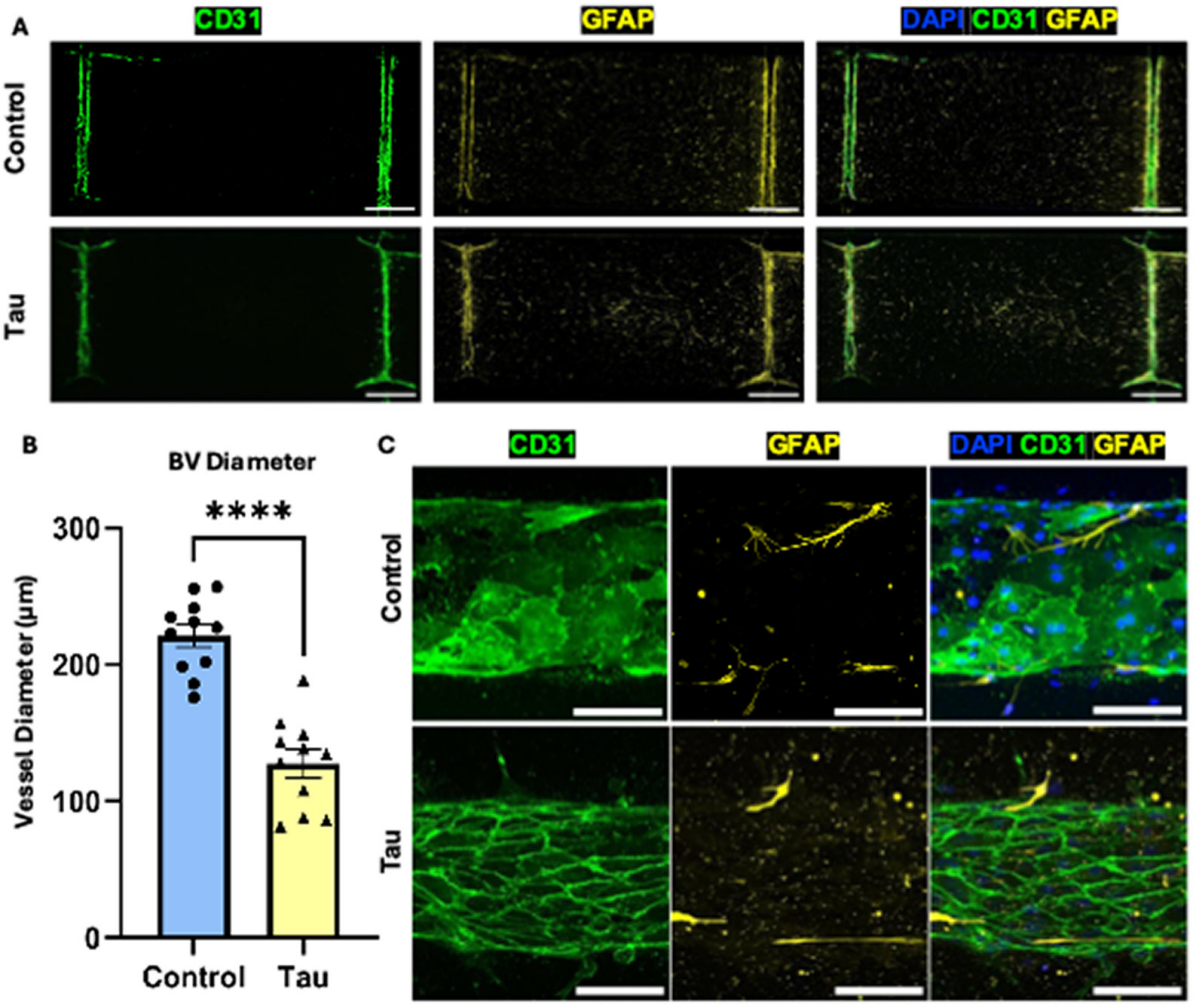
Tau-induced astrocytic reactivity drives blood vessel constriction. (a) Immunofluorescence images displaying the endothelial marker CD31 (green) and astrocytic marker GFAP (yellow) in the control and tau-treated groups within the glymphatics-on-chip platform. DAPI (blue) marks nuclei. (b) Quantification of BV diameter in the control and tau-treated groups (N = 11). (c) Immunofluorescence images of endothelial cells (CD31, green) and astrocytes (GFAP, yellow). Merged views depict astrocyte-BEC interactions and structural alterations in the tau-treated group. Scale bars: 1 mm (a), 200 *μ*m (c). Statistical significance: ^****^(p < 0.0001).

To investigate whether p-tau affects vascular morphology, we analyzed BV structure in the glymphatics-on-chip device after tau exposure. Immunofluorescence staining of CD31 and GFAP revealed that tau-treated chips exhibited markedly irregular, constricted blood vessels compared to controls, which retained smooth, cylindrical lumens [Fig. 2(a)]. Quantitative analysis demonstrated a significant reduction in BV diameters under tau conditions [Fig. 2(b)]. High-resolution imaging showed that these constrictions happened at the regions where astrocytes were in close contact with the vascular surface, in conjunction with increased GFAP expression and CD31 junctional localization [Fig. 2(c)], suggesting a mechanical interaction between reactive astrocytes and endothelial structures. These findings point to a previously underappreciated role of astrocytic reactivity in regulating vascular tone, with tau-induced changes promoting vasoconstriction via direct glial-vascular contact.

### C. Tau impairs glymphatic solute clearance and disrupts interstitial flow symmetry

**FIG. 3.**
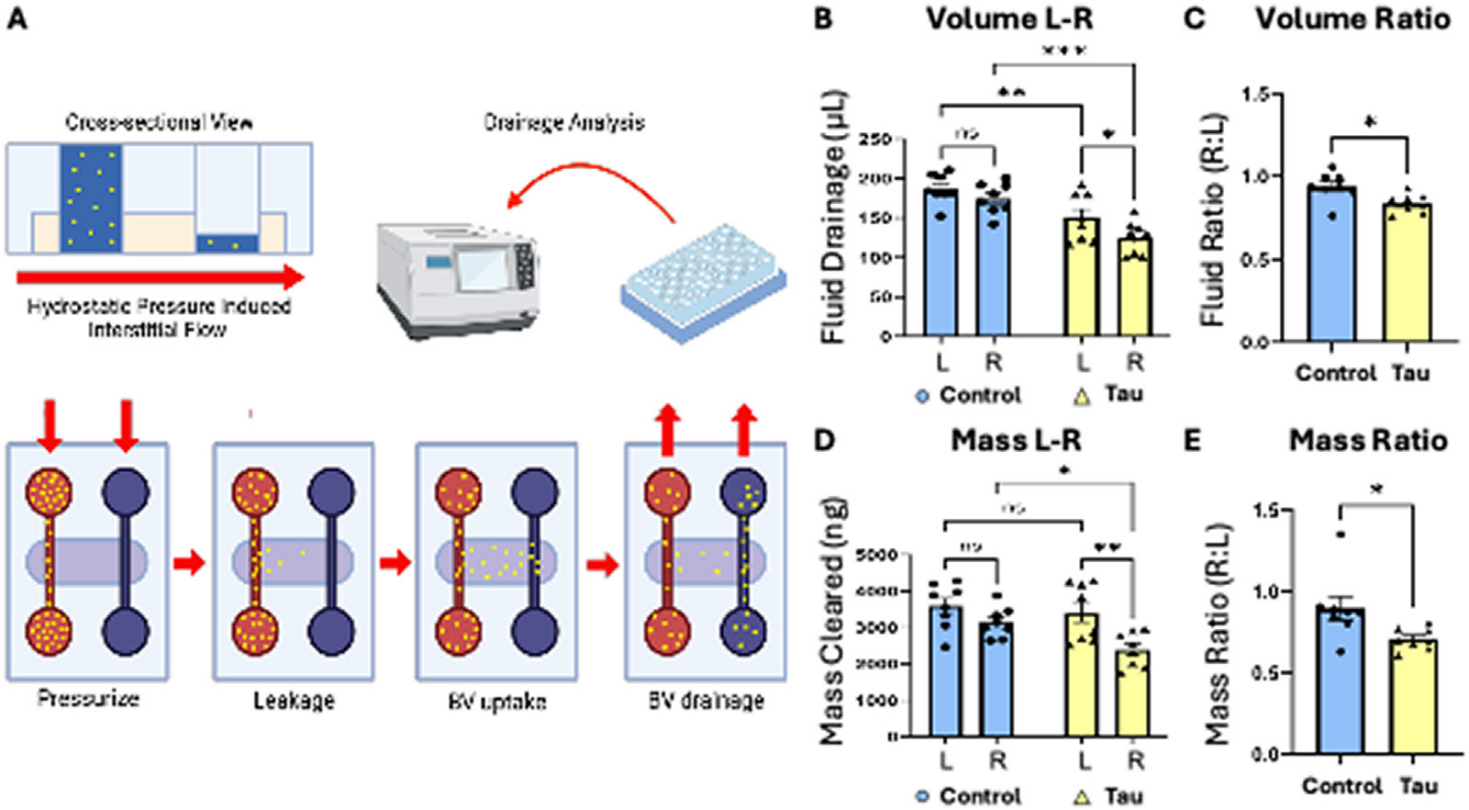
Tau impairs glymphatic solute clearance and disrupts interstitial flow symmetry. (a) A schematic sequence outlining the interstitial fluid drainage experiments in the glymphatics-on-chip system. The process includes hydrostatic pressurization in the left side channel, leakage into the adjacent ECM bulk, uptake by engineered BVs, and final collection of the fluid in four reservoirs after 16 h to calculate fluid/mass drainage. (b) A bar graph displaying fluid drainage (*μ*L) from the left and right sides of the chip (N = 8). (c) A bar graph illustrating the fluid drainage ratio between the left and right channels under control and tau-treated conditions, used as a measure of flow symmetry across the matrix. A value near 1.0 reflects balanced drainage, while a deviation from 1.0 indicates asymmetric flow. (N = 8). (d) A bar graph quantifying mass clearance (150 kDa dextran) from the left and right drainage areas (N = 8). (e) A bar graph depicting the mass clearance ratio between control and tau groups, similarly used to assess the spatial distribution of solute transport. (N = 8). Statistical significance: ^*^(p < 0.05), ^**^(p < 0.01), and ^***^(p < 0.001); ns = not significant.

To recapitulate cerebral interstitial fluid drainage (∼1.7 *μ*m/s) in the process of glymphatic clearance, we applied a differential hydrostatic pressure (∼0.47 mm Hg) across the chip and quantified both fluid movement and macromolecular solute clearance [Fig. 3(a)]. Drained fluid in the right-side reservoirs was collected and analyzed to determine fluid and mass drainage. Under tau-treated conditions, total fluid volume drained from left to right reservoirs was significantly decreased [Fig. 3(b)], and flow symmetry—measured as the right-to-left drainage ratio—was disrupted [Fig. 3(c)]. The fluid volume ratio—an indicator of drainage symmetry—has a value of 1.0 when the flow is perfectly balanced. However, the ratio was significantly reduced in the tau group compared to the control [Fig. 3(c)]. Moreover, clearance of 150 kDa FITC-labeled dextran was also significantly reduced in tau-treated devices [Figs. 3(d) and 3(e)], indicating impaired glymphatic solute transport. These effects were not due to channel occlusion or system failure, as luminal perfusion was maintained in all conditions on a rocking platform throughout experiments. Collectively, these results demonstrate that p-tau impairs both the magnitude and spatial balance of interstitial fluid flow, recapitulating hallmark features of glymphatic dysfunction seen in AD.

### D. Astrocytic contractility mediates tau-induced vasoconstriction and glymphatic dysfunction

**FIG. 4.**
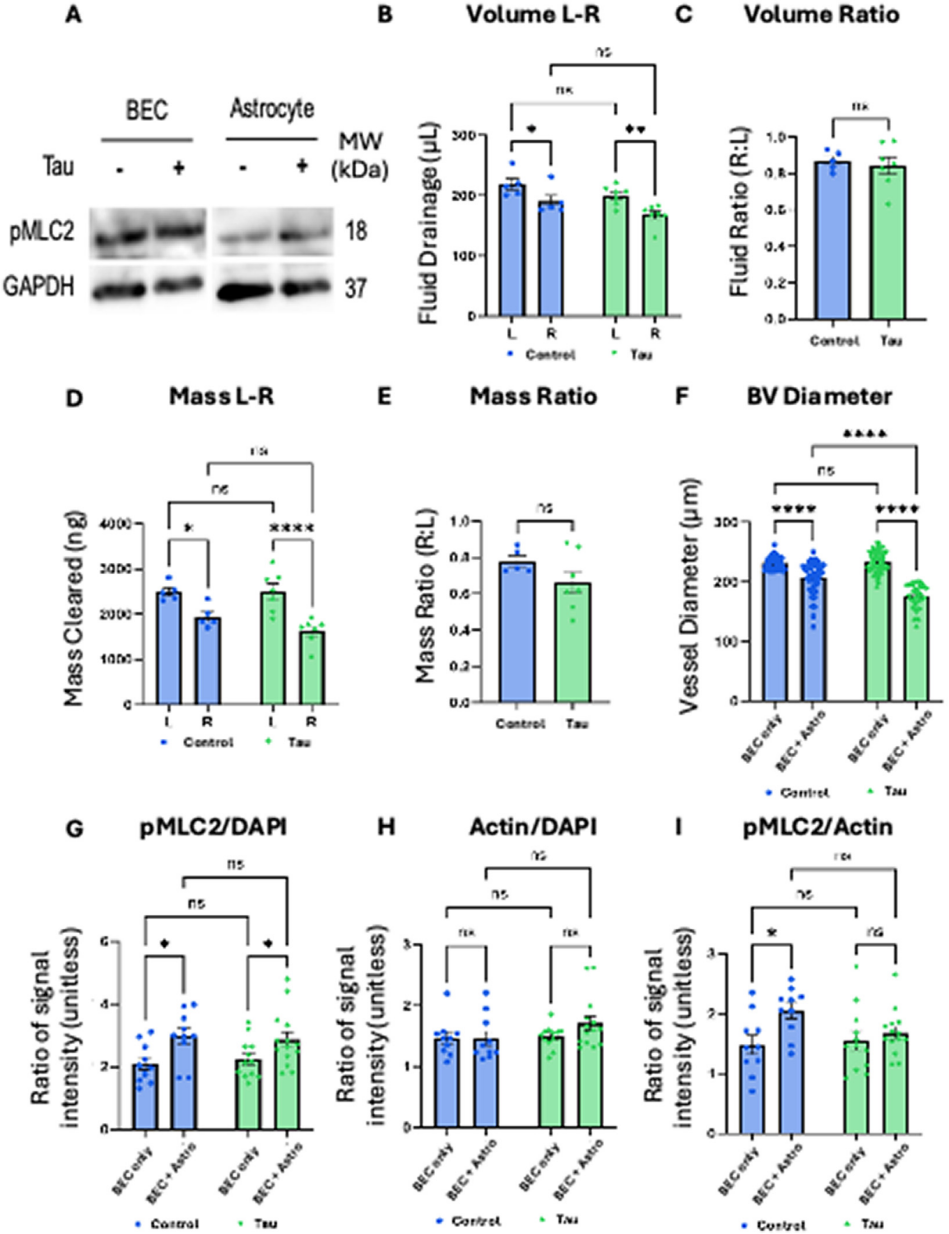
Astrocytic contractility mediates tau-induced vasoconstriction and glymphatic dysfunction. (a) Western blot (WB) analysis of phosphorylated myosin light chain 2 (pMLC2) in BECs and astrocytes with or without tau. GAPDH serves as a loading control. (b) A bar graph displaying fluid drainage (*μ*L) from the left and right sides of the chip in BEC monoculture (without astrocytes) with or without tau (N = 5–7). (c) A bar graph illustrating the fluid drainage volume ratio between the left and right sides of the chip in BEC monoculture with or without tau (an ideal ratio of 1.0 representing equilibrium) (N = 5–7). (d) A bar graph quantifying mass clearance (150 kDa dextran) from the left and right sides of the chip in BECs monoculture with or without tau (N = 5–7). (e) A bar graph depicting the mass clearance ratio of the chip in BECs monoculture with or without tau (N = 5–7). (f) A bar graph showing blood vessel (BV) diameter across the four groups in order: (1) BEC monoculture without tau, (2) BEC/astrocyte coculture without tau, (3) BEC monoculture with tau, and (4) BEC/astrocyte coculture with tau (standard tau-mediated AD model) (n = 25–35, N = 5–7). (g) A bar graph of pMLC2 signal intensity normalized to DAPI in BVs across the four groups (n = 25–35, N = 5–7). (h) A bar graph of actin intensity normalized to DAPI in BVs across the four groups (n = 25–35, N = 5–7). (i) A bar graph of pMLC2 intensity normalized to actin in BVs across the four groups (n = 25–35, N = 5–7). Statistical significance: ^*^(p < 0.05), ^**^(p < 0.01), and ^****^(p < 0.0001); ns = not significant.

To determine whether tau affects endothelial cells directly or acts through astrocytes, we assessed their contractile activities by measuring the expression level of phosphorylated myosin light chain II (pMLC2). Western blot analysis revealed no change in pMLC2 expression following tau exposure in BECs alone [Fig. 4(a)]. In contrast, astrocytes exhibited strong upregulation of pMLC2, indicating heightened actomyosin contractility [Fig. 4(a)]. This suggests that astrocytes, rather than BECs, are the primary responders to tau in our model. Indeed, in monoculture drainage assays using only BECs, tau did not affect fluid volume, drainage symmetry, or mass clearance [Figs. 4(b)–4(e)]. Vessel diameter remained unchanged in BEC monocultures, regardless of tau presence. However, when astrocytes were present, tau caused significant vessel narrowing [Fig. 4(f)], confirming astrocyte-specific mediation of vasoconstriction. To clarify the mechanism underlying vascular dysfunction, we distinguish between two related but mechanistically distinct phenomena: astrocyte contractility and astrocyte-driven vasoconstriction. Astrocyte contractility is the cell-intrinsic generation of cytoskeletal tension, primarily mediated by actomyosin dynamics. In contrast, astrocyte-driven vasoconstriction describes the downstream tissue-level effect, in which contractile forces are transmitted through perivascular end-feet, leading to physical narrowing of adjacent blood vessels. This conceptual distinction provides a mechanistic link between p-tau exposure and vascular impairment via a glial-specific pathway. To validate this link, we quantified pMLC2, actin filaments, and their ratio within the vascular compartment. Although actin intensity remained stable [Fig. 4(h)], both pMLC2 levels and the pMLC2-to-actin ratio were significantly elevated in tau-treated co-cultures [Figs. 4(g)–4(i)]. These findings support that tau-induced astrocyte hypercontractility directly contributes to vessel constriction and impaired glymphatic function.

Taken together, tau exposure enhances astrocytic contractility, which in turn drives vessel narrowing and impairs solute clearance without directly affecting endothelial contractility. Tau-induced astrocytic hypercontractility thus emerges as a mechanistically distinct and therapeutically relevant contributor to glymphatic dysfunction and cerebrovascular impairment in AD.

### E. Inhibition of non-muscle myosin II reverses tau-induced vascular and glymphatic defects

**FIG. 5.**
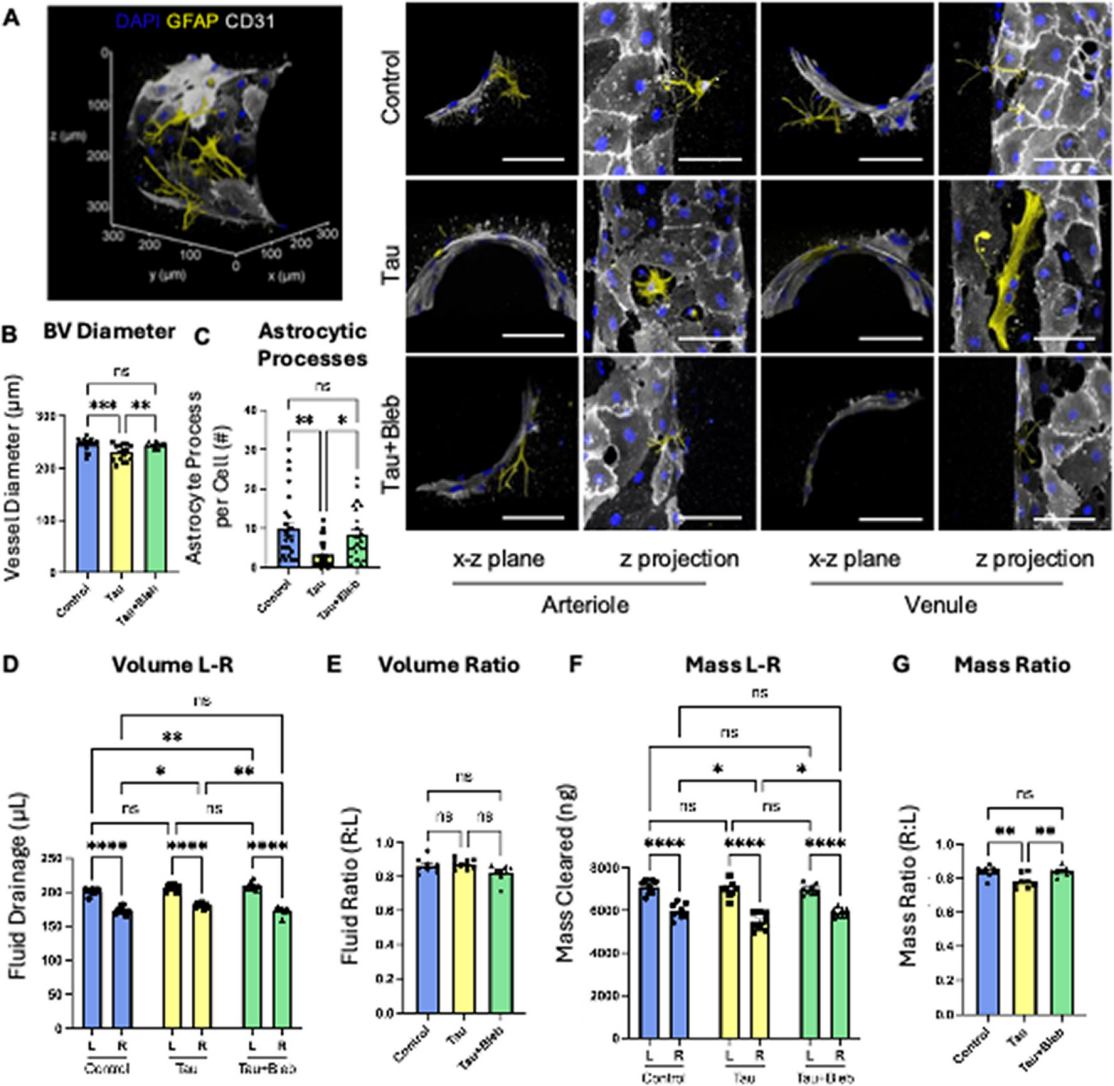
Inhibition of non-muscle myosin II reverses tau-induced vascular and glymphatic defects. (a) Immunofluorescence images showing astrocytic marker GFAP (yellow), endothelial marker CD31 (grey), and nuclei (DAPI, blue) in control, tau-treated, and tau + blebbistatin-treated groups. Zoomed views highlight the x–z plane and maximum projection of the z-stack images of arterioles and venules. (b) A bar graph of BV diameter across control, tau-treated, and tau + blebbistatin-treated groups (N = 8). (c) A bar graph showing the number of astrocytic processes per cell across the experimental groups (N = 8). (d) A bar graph displaying fluid drainage (*μ*L) from the left (L) and right (R) sides of the chip across the three groups (N = 8). (e) A bar graph illustrating the fluid drainage ratio between the left and right sides across the three groups (N = 8). (f) A bar graph quantifying mass clearance (150 kDa dextran) from the left (L) and right (R) drainage areas across the three groups (N = 8). (g) A bar graph depicting the mass clearance ratio across control, tau-treated, and tau + blebbistatin-treated groups (N = 8). Statistical significance: ^*^(p < 0.05), ^**^(p < 0.01), ^***^(p < 0.001), and ^****^(p < 0.0001); ns = not significant. Scale bars, 100 *μ*m (a).

We next tested whether astrocytic contractility could be therapeutically targeted using blebbistatin, a selective inhibitor of non-muscle myosin II. Immunofluorescence images revealed key structural differences of the NVU under control and tau conditions. Under control conditions, astrocytes exhibited a stellate morphology with well-organized processes extending to cover BVs [Fig. 5(a)]. These structures provided moderate support, resembling the trabecular meshwork, and maintained healthy glymphatic clearance. In contrast, astrocytes exposed to tau adopted a hypertrophic, tufted morphology with fewer, disorganized processes and tighter vessel association—characteristics indicative of a reactive state. Despite the reduced number of processes [Fig. 5(c)], tau-treated astrocytes showed increased total coverage along the vessel wall, potentially contributing to excessive mechanical tension and reduced vessel diameter. These structural changes were pronounced across the ECM bulk, particularly in the proximity of arterioles and venules [Fig. 5(a)], where astrocytes exert the most substantial influence on vascular tone and reduced mass transport and clearance. Treatment with blebbistatin restored astrocyte morphology from hypertrophic to stellate [Fig. 5(a)], increased the number of astrocytic processes [Fig. 5(c)], and normalized BV diameters [Fig. 5(b)]. Fluid drainage volume and symmetry remained unchanged across groups [Figs. 5(d) and 5(e)], but mass clearance and distribution significantly improved following blebbistatin treatment [Figs. 5(f) and 5(g)], suggesting that astrocyte-driven vascular narrowing selectively impairs solute transport while sparing bulk flow. These findings confirm that tau-induced glymphatic dysfunction is mediated by astrocytic contractility and that targeting this pathway can restore vascular tone and clearance function, highlighting its potential as a therapeutic avenue in AD.

## III. DISCUSSION

Understanding the cellular mechanisms underlying glymphatic dysfunction in AD remains a major challenge in neurodegeneration research due to the complex cellular network constituent of a wide range of diverse cell types in the brain.^46^ Here, we employed a human glymphatics-on-chip platform that incorporates engineered vasculatures and primary cortical astrocytes to study tau-induced effects on vascular regulation and solute clearance. Our findings uncover a glial-specific mechanism in which p-tau promotes astrocytic hypercontractility, leading to vasoconstriction and impaired interstitial fluid clearance. These results advance our mechanistic understanding of early AD pathophysiology and introduce astrocyte contractility as a modifiable target for therapeutic intervention. The hypertrophic and contractile transformation of astrocytes in response to p-tau exposure mirrors key features of reactive gliosis commonly seen in AD.^47,48^ By using a physiologically relevant tau phosphorylation strategy (PKA and SAPK4),^18^ we generated tau species bearing disease-associated epitopes while avoiding the artifacts of synthetic aggregation methods such as heparin induction.^38^ This approach enabled us to model early tau pathology without inducing cytotoxicity or neurofibrillary tangle formation.^39^

Astrocytes exposed to p-tau developed tufted morphologies and exhibited increased actin area and pMLC2 expression, confirming their transition into a reactive and contractile state. Phosphorylated myosin light chain II is a well-established marker of actomyosin-driven cytoskeletal contractility and reflects the activation of non-muscle myosin II.^49^ It functions as a sensitive indicator of intracellular tension in both endothelial cells and astrocytes.^50,51^ In the context of AD, pMLC2 can detect early biomechanical changes that precede overt vascular barrier dysfunction. Given the anatomical localization of astrocytic end-feet around cerebral blood vessels,^52^ elevated pMLC2 levels in tau-treated astrocytes underlie a mechanistic link between pathogenic tau and increased astrocytic contractility.^53,54^ Notably, tau treatment did not affect all astrocytes uniformly, consistent with our use of a sub-cytotoxic tau concentration (1 *μ*M) designed to model the early stage of AD-associated hypoperfusion. Morphological changes were more pronounced in the 3D vascularized chip than in monoculture, suggesting that vascular cues and fluid dynamics amplify tau-induced remodeling. Interactions with endothelial cells and directional interstitial flow likely promote astrocytic polarization, leading to substantial cytoskeletal reorganization. Consistent with prior studies linking tau pathology to glial activation and hypertrophy,^55–57^ these alterations reflect a pathological shift in astrocyte morphology and behavior, reinforcing the critical role of astrocytes as mediators of AD progression. Importantly, our data show that tau-induced astrocytic remodeling leads to physical constriction of engineered blood vessels in our glymphatics-on-chip model. This vasoconstriction was not observed in endothelial monocultures, nor did p-tau directly activate pMLC2 in BECs, indicating a glial-specific effect. The constricted vessels, particularly in astrocyte-dense regions, suggest that tau-exposed astrocytes mechanically restrict vessel diameter through their perivascular processes. This aligns with previous *in vivo* reports of glial-mediated vascular tone regulation and, meanwhile, provides direct evidence linking astrocytic biomechanics to tau pathology.^16,31,36,58^

Glymphatic dysfunction in AD has typically been attributed to amyloid burden or vascular stiffness. However, our model demonstrates that astrocytic contractility alone, triggered by tau, is sufficient to impair interstitial fluid drainage. Mass clearance of 150 kDa dextran was significantly reduced in tau-treated chips, while fluid volume drainage remained relatively stable. This suggests a selective impairment in solute transport, which could exacerbate the accumulation of neurotoxic waste products such as amyloid-β and tau aggregates in the brain parenchyma. These findings are consistent with clinical imaging studies showing impaired glymphatic function and hypoperfusion in early AD and mild cognitive impairment.^1,2,31,36^ In contrast to prior studies that employed chronic, late-stage tau in endothelial-centered systems to study vascular breakdown, our approach captured early-stage, astrocyte-specific responses. This distinction was critical in revealing that tau-induced glymphatic dysfunction and vessel constriction can arise through astrocytic contractility alone, independent of overt endothelial damage. Our model thus provides a disease-specific platform for probing glial contributions to neurovascular impairment and establishes a tractable system for investigating early and potentially reversible mechanisms in AD progression.

Aging is the most substantial risk factor for the late-onset neurodegenerative diseases, including AD, contributing to progressive declines in vascular compliance, glymphatic clearance, and glial regulation.^2,59^ While traditional therapeutic strategies have focused on the root cause of neurodegeneration by targeting upstream pathologies such as Aβ and tau accumulation,^60^ recent approaches have shifted toward addressing the functional consequences of aging-related dysfunction and decelerating disease progression. Our findings demonstrate that pharmacological inhibition of non-muscle myosin II with blebbistatin directly reverses tau-induced astrocyte hypercontractility, restores blood vessel diameter, and improves glymphatic solute clearance, representing a cell-specific correction of biomechanical dysfunction in a disease-relevant setting. While most AD therapies have focused on neuronal or microglial mechanisms,^22,61^ our data suggest that targeting downstream consequences of tau aggregation, such as biomechanical dysregulation in astrocytes, may offer a complementary approach to restore lost function. These findings raise the possibility that blebbistatin or similar drugs could be repurposed to address functional deficits in AD, particularly by targeting impaired glymphatic clearance and vascular tone regulation. Given the emergence of astrocyte-specific delivery platforms,^62^ including AAVs and nanoparticle systems,^63^ and the broader roles of non-muscle myosin II in glial reactivity across diverse brain cell types,^64^ this pathway may represent a viable therapeutic strategy for targeting neurovascular dysfunction in neurodegenerative diseases.

While our glymphatics-on-chip model provides a robust platform to dissect glial–vascular interactions under disease-relevant conditions, it also presents some limitations. The model does not fully recapitulate the complex cellular and biophysical interactions present *in vivo*, such as immune cell contributions,^65^ systemic blood flow, or pressure dynamics.^66^ Future iterations of the platform should include additional NVU components, such as microglia and pericytes, to better replicate the *in vivo* glymphatic system. In addition, chronic exposure models and dose–response studies are needed to characterize long-term effects of tau and test therapeutic durability. Finally, while we focused on cortical astrocytes due to their relevance in early tau deposition,^13,67^ astrocyte heterogeneity remains an important consideration.^68^ Astrocytes derived from other brain regions, such as the hippocampus or brainstem, may exhibit distinct contractile behaviors or tau responses.^69,70^ Comparative studies across astrocyte subtypes could further delineate region-specific vulnerabilities in AD.

In summary, this study reveals that phosphorylated tau induces astrocyte-driven vasoconstriction and glymphatic impairment through increased cytoskeletal contractility. Targeting glial biomechanics—particularly non-muscle myosin II—represents a novel strategy to restore perivascular function and solute clearance in Alzheimer's disease. Our findings underscore the importance of astrocyte mechanics in neurodegenerative disease and provide a human-relevant platform for future therapeutic discovery.

## IV. METHODS

### A. Cell culture

Human astrocytes (ScienCell, #1800) derived from the cerebral cortex were cultured in complete astrocyte media (ScienCell, #1801) containing FBS and astrocyte growth factors. Primary human dermal blood endothelial cells, d-HMVEC-BlNeos (Lonza), were maintained in EBM-2 supplemented with EGM-2 MV Single Quots (Lonza). Media changes for both cell types occurred every three days. Cells were incubated at 37 °C with 95% humidity and 5% CO_2_ in a cell culture incubator.

### B. Tau hyperphosphorylation

Tau hyperphosphorylation method was adapted from Meng *et al.*^18^ Recombinant full-length tau protein (0N4R) was utilized in our phosphorylation studies. A reaction mixture containing 25 mM Tris-HCl buffer (pH 7.4) was prepared, with the addition of 0.1 mM EGTA, 2 mM AEBSF protease inhibitor, 10 mM magnesium acetate, and 2 mM ATP to support kinase activity. To initiate phosphorylation, catalytic subunits of cAMP-dependent protein kinase A (PKA) were introduced at a proportion of 0.018 units per nanomole of tau. The mixture underwent incubation at 30 °C for 24 h for initial phosphorylation, followed by adding an additional kinase SAPK4 (MAPK12) and maintaining the same conditions for a further 24 h to enhance phosphorylation. The phosphorylated tau was then aliquoted at a concentration of 40 *μ*M and stored at −80 °C for subsequent analysis.

### C. 3D cell-suspended ECM plate model

Prior to the introduction of the ECM containing suspended astrocytes, the glass surface of the eight-well glass-bottomed plate (MatTek Life Sciences) was treated with oxygen plasma, 0.01% poly-L-lysine (Sigma-Aldrich), and 1% glutaraldehyde (EMS) to reduce surface hydrophobicity. The plates were rinsed overnight with distilled water and UV-treated before the addition of 200 *μ*l of ECM per well. The ECM was composed of 2.5 mg/ml collagen I (Corning), 1.25 mg/ml thiol-treated hyaluronic acid (HyStem kit, Advanced Biomatrix), 0.26 mg/ml human plasma fibronectin (Sigma-Aldrich), 10X phosphate-buffered saline (PBS, Gibco), 1 N sodium hydroxide (NaOH, EMS), and astrocyte media containing human astrocytes at a density of 1 × 10^6^ cells/ml. For experiments incorporating hyperphosphorylated tau, it was included in the ECM at a specified concentration. After 1 h of incubation in the cell culture incubator, additional astrocyte media was added. Post a day of static culture, the plates were placed on a rocker inside the incubator.

### D. Chip model

Following the methods outlined in Soden *et al.*,^24^ devices were constructed using silicon photolithography to create the mold pattern. Polydimethylsiloxane (PDMS) was combined at a 10:1 ratio with its curing agent (Sylgard) and degassed under vacuum. The mixture was then cast onto the molds and cured at 80 °C overnight. Once cured, the molds were removed and shaped with razors and biopsy punches. For device assembly, the PDMS molds were bonded to 1.5 mm thick glass coverslips (EMS) using oxygen plasma treatment in a PE-25 Plasma Cleaner (Plasma Etch Inc., NV, USA) at 18 W for 2 min. This first plasma step served to create permanent covalent bonding between the PDMS and glass. After bonding, devices were thermally cured again at 80 °C for at least 10 min to ensure structural stability. To reduce PDMS hydrophobicity and facilitate ECM channel filling, a second oxygen plasma treatment was performed on the fully assembled devices using the same power setting (18 W) for 5 min to improve surface wettability and is followed by sequential coating with 0.01% poly-L-lysine (Sigma-Aldrich), 1% glutaraldehyde (EMS), and an overnight rinse with distilled water. Acupuncture needles with a 0.25 *μ*m diameter (Lhasa OMS) were sterilized in ethanol, coated with 1% bovine serum albumin (BSA) in PBS, and inserted into the devices. After drying and UV sterilization, the ECM enriched with astrocytes (identical to that in the 3D cell-suspended ECM model) was added, and the devices were incubated overnight in a static cell culture incubator. The following day, the needles were removed, the devices sealed with vacuum grease (EMS), and refreshed with astrocyte media. The devices were then placed on a gravity rocker overnight. Subsequently, BECs were seeded at a concentration of 500 000 cells/ml in complete microvascular endothelial media, alternating the orientation of the devices before subjecting them to shear and radial stresses of approximately 4 and 2 dyne/cm^2^, respectively, on a gravity rocker.

### E. Immunofluorescence imaging

Post-fixation, both well plates and chips were prepared identically for immunofluorescence imaging. Samples were fixed using 4% paraformaldehyde (PFA, EMS) in PBS for well plate samples and in EBM-2 for co-culture chips for 30–40 min, followed by three PBS washes and overnight soaking in PBS at 4 °C. A 0.3% solution of Triton X-100 in PBS was prepared to permeabilize the cells. This solution was added to each sample for 45–60 min at room temperature with agitation. Following this, samples were blocked in 3% BSA in PBS, treated with primary antibodies including mouse anti-CD31 (1:100, Dako), goat anti-GFAP (1:100, abcam), or rabbit anti-pMLC (1:100, Cell Signaling) in 3% BSA, washed, and then incubated with secondary antibodies (1:500, all from ThermoFisher Scientific), phalloidin (1:200, ThermoFisher Scientific), and DAPI (1:500, ThermoFisher Scientific). After a final wash, the samples were imaged using a Leica DMi8 confocal light microscope.

### F. Image analysis

Astrocyte morphology, highlighted by F-actin staining with phalloidin, was randomly selected from each image for assessment. Using ImageJ, the F-actin structures were manually traced, and measurements of area, circularity, and roundness were obtained. For the initial analysis of the BV diameter, each chip schematic was represented by 10× magnification tile scan images. In these images, ten lines were drawn across both the left and right channels throughout the ECM reservoir to measure BEC diameter consistently. Measurements were made using ImageJ by manually tracing lines across the diameter in each identified channel. For the following BV measurements, 20× magnification tile scan images of only the BV were collected. Using ImageJ, a quadrilateral perimeter of each vessel was drawn using the polygonal selection tool, and mean signal intensity was measured for the region of interest (ROI). Five diameter measurements were made with the line selection tool across the CD31 channel of each image. All images were blinded to ensure objectivity. To quantify the number of astrocytic processes, 40× z-stack images of BVs were taken and reconstructed in the Leica Application Suite (LAS) X; astrocytes around the vessels were identified, and the number of astrocytic processes was obtained by manually counting the branches in the 3D viewer.

### G. Glymphatic drainage assay

The glymphatic drainage function was assessed using fluorescently labeled FITC dextran (150 kDa, ThermoFisher). Dextran was introduced into the microfluidic devices via media reservoirs linked to the BEC channels. To induce interstitial fluid flow, hydrostatic pressure differentials were applied, with 400 *μ*l of complete microvascular endothelial cell media containing dextran added to the two left media reservoirs (200 *μ*l each) and 40 *μ*l to the right media reservoirs (20 *μ*l each). The devices were placed on a rocking platform to maintain luminal flow within the channels. After 16 h, fluids from both reservoirs were collected separately, and the volumes were measured individually. To estimate the pressure gradient driving unidirectional interstitial fluid flow, we calculated the height difference (Δh) between the media reservoirs using the following equation:

$$\Delta h=\frac{\Delta V}{\pi r^{2}}=\frac{V_{200}-V_{20}}{\pi r^{2}}=\frac{200\;\mu l-20\;\mu l}{\pi {\left(3\;\text{mm}\right)}^{2}}=6.37\;\text{mm}=0.006\;37\;m,$$ (1)

where ΔV is the difference in the initial media volumes between the left and right reservoirs and *r* is the reservoir radius (3 mm). The resulting hydrostatic pressure difference was then calculated as

$$\Delta P=\rho g\Delta h=1000\frac{\text{kg}}{m^{3}}\times 9.81\frac{m}{s^{2}}\times 0.006\;37\;m=62.5\;\text{Pa}\;\approx 0.469\;\text{mmHg},$$ (2)

where ρ is the fluid density and g is the acceleration due to gravity. To estimate the interstitial fluid flow rate, we applied the following equation:

$$v=\frac{\Delta V}{At}=\frac{175\;\mu l-40\;\mu l}{\left(450\;\mu m\right)\left(3\;\text{mm}\right)\left(16\;h\right)}=1.7\;\mu m/s,$$ (3)

where ΔV is the difference in the average volume taken from the right reservoirs after and before drainage, A is the cross-sectional area of the ECM chamber, with a height of 450 *μ*m and width of 3 mm, and t is the drainage duration time. A schematic of the whole chip layout, channel dimensions, and hydrostatic setup is provided in the supplementary material. Concentrations of the labeled dextran either retained in the left reservoirs or drained to the right reservoirs were determined using the SpectraMax M2 (Molecular Devices). For the treatment group, blebbistatin (Sigma, 10 *μ*M) was added to the media a day before the drainage assay and treated for 24 h.

### H. Western blot analysis

Confluent BECs or astrocytes cultured in well plates were treated with or without p-tau. After 24 h, cells were rinsed with cold PBS and lysed in Pierce™ IP Lysis Buffer (ThermoFisher) with protease and phosphatase inhibitors (Sigma). Clarified lysates were equalized for protein content, mixed with 1× Laemmli SDS Sample Buffer (Boston Bioproducts), and then boiled at 95 °C for 10 min. The lysate proteins were fractionated by SDS-PAGE and transferred to a PVDF membrane. After blocking, the membrane was incubated with primary antibodies, including GAPDH (1:1000, Cell Signaling), and pMLC2 (1:1000, Cell Signaling), followed by secondary antibody incubation with HRP-linked anti-rabbit IgG antibody (1:1000, Cell Signaling). The protein bands were visualized by exposure to the Clarity™ Western ECL Substrate (BioRad), and the chemiluminescent HRP was detected by the Amersham Imager 680 (Cytiva). The acquired images were adjusted for brightness and contrast using ImageJ.

### I. Statistics

A two-sided, unpaired Student's t-test was used to analyze astrocyte morphology, vessel diameter, and fluid and mass drainage ratio with two independent groups. For group analyses, one-way analysis of variance (ANOVA) with Tukey's post hoc test was used for the analysis of vessel diameter, drainage ratio, and number of astrocytic processes with blebbistatin treatment. Two-way ANOVA with Tukey's post hoc test was used to analyze fluid and mass drainage, comparing left and right and drainage experiments with four groups. The p-values and sample numbers (technical replicates, N) are detailed in the figure legends. Statistical analysis was conducted in GraphPad Prism 10. Significant p-values were defined as follows: ns, not significant; ^*^P < 0.05; ^**^P < 0.01; ^***^P < 0.001; and ^****^P < 0.0001. Data are means ± SEMs.

## SUPPLEMENTARY MATERIAL

See the supplementary material figure for a schematic of the glymphatics-on-chip layout, channel dimensions, and hydrostatic setup.

## Data Availability

The data that support the findings of this study are available from the corresponding author upon reasonable request.
